# Therapeutic effect of an MRGPRX2/MRGPRB2 antagonist on LL-37-induced rosacea-like inflammation in mice

**DOI:** 10.1007/s00011-025-02144-y

**Published:** 2025-11-26

**Authors:** Billy Kwok Chong Chow, Ye Gi Choi, Trevor K. Wong, Shaik Abdullah Nawabjan, Kesang Li, Mukesh Kumar

**Affiliations:** 1 Ningbo No.2 Hospital, Ningbo, Zhejiang China; 2 Guoke Ningbo Life Science and Health Industry Research Institute, Ningbo, Zhejiang China; 3 School of Biological Sciences, The University of Hong Kong, Pok Fu Lam Road, Pok Fu Lam, Hong Kong China; 4 Faculty of Health Sciences, McMaster University, Hamilton, ON L8S 4L8 Canada

**Keywords:** Mast cells, MRGPRX2, Rosacea, Allergy, Inflammation, Small molecule antagonist

## Abstract

**Introduction:**

Rosacea is a chronic inflammatory skin disorder characterized by symptoms like itching, redness, and impaired skin barrier function. Mast cell activation plays a crucial role in its pathogenesis. Recent evidence shows higher expression of mast cell receptor MRGPRX2/MRGPRB2 in rosacea patients’ skin tissues and its potential as a novel drug target. We evaluated the therapeutic effect of a novel small-molecule MRGPRX2/MRGPRB2 antagonist in a mouse model of rosacea and itch.

**Methods:**

The therapeutic effects of GE1111 were evaluated in vivo on wildtype and MRGPRB2 knock-out mice with LL-37-induced rosacea. Serum MCP-1 level and histochemistry measured inflammation and mast cell degranulation in skin tissue. Functional in vitro cell culture assays were developed using MRGPRX2/MRGPRB2 agonist LL-37, mast cells, keratinocytes, and macrophage cell lines.

**Results:**

LL-37-treated mice showed redness, increased serum MCP-1, and epidermal thickness of skin tissue, while these changes were absent in LL-37-treated MRGPRB2 knock-out mice. Treatment with GE1111 reduced rosacea symptoms, epidermal thickness, and serum MCP-1 levels. GE1111 protected tight junction protein expression and reduced mast cell degranulation and inflammatory cytokine gene and protein expression in skin lesions. GE1111 treatment reduced the number and duration of itch in the compound 48/80 induced itch model. In vitro evidence showed GE1111’s mechanism by inhibiting inflammatory interaction of mast cells with keratinocytes and macrophages.

**Conclusion:**

GE1111 showed promising therapeutic effects in rosacea via targeting interactions between mast cells, keratinocytes, and macrophages and inhibiting inflammatory cytokines. These findings open possibilities for developing MRGPRX2/MRGPRB2 antagonists as novel treatments for rosacea.

## Introduction

Rosacea is a chronic inflammatory skin condition that primarily affects the central face and is characterised by persistent erythema, papules, pustules, itching and telangiectasia [1, 2]. Despite its high global prevalence of 5–10% and its impact on quality of life, the pathogenesis of rosacea remains poorly understood [3–5]. Moreover, the current treatment options for rosacea focus mainly on managing the clinical symptoms rather than targeting the underlying mechanisms [6]. Topical and systemic antibiotics and anti-inflammatory agents are commonly prescribed, but their efficacy is limited and often associated with adverse effects. Therefore, novel therapeutic strategies are urgently needed to target the pathogenic mechanisms involved in rosacea.

The multifactorial nature of rosacea involves a complex interplay of genetic, environmental, and immune factors. It is crucial to understand the underlying mechanism and identify novel druggable targets. One key player in the pathogenesis of rosacea is the antimicrobial peptide LL-37, which is upregulated in the skin of rosacea patients [7–9]. LL-37 is known to activate various immune cells, including mast cells (MCs), releasing pro-inflammatory mediators and perpetuating the inflammatory dermatological response in rosacea [10, 11]. Several studies have reported the MCs degranulation activity of LL-37, which might play a crucial pathogenic role in rosacea [5, 12, 13].

Recently, the Mas-related G protein-coupled receptor X2 (MRGPRX2) and its mouse orthologue MRGPRB2 have emerged as a potential therapeutic target for non-IgE mediated MC degranulation and inflammatory skin conditions [5, 14–16]. MRGPRX2 expression in mast cells is well-documented, whereas its presence in basophils and dorsal root ganglia remains a topic of debate. Additionally, over 20 peptides and non-peptide ligands have been identified as activators of the MRGPRX2/B2 receptor, indicating its diverse functional role in immune responses [5]. LL-37 is now established as an MRGPRX2/B2 agonist, which showed MCs degranulation activity [17]. Our group has recently developed novel small molecule MRGPRX2 antagonists, which showed a significant anti-allergic activity via inhibiting MRGPRX2/B2 mediated MC degranulation and inflammatory cytokine release [18]. We have tested the efficacy of MRGPRX2 antagonists against acute allergic response and systemic anaphylaxis in mouse models. Other studies targeting MRGPRX2 also showed a promising therapeutic potential of MRGPRX2/B2 antagonists to develop as a novel class of antiallergic drugs [17–19].

In the present study, we aimed to investigate the therapeutic effect of one of the potent MRGPRX2/B2 antagonists, GE1111, by using an in vivo disease model and a battery of in vitro functional assays. We used LL-37 as a prototype MRGPRX2/B2 agonist to induce rosacea symptoms in mice and modulate the immune microenvironment in vitro. We evaluated the effect of GE1111 on an LL-37-induced rosacea-like inflammation mouse model and compound 48/80 (C48/80) induced itch mouse model by incorporating wild-type and MRGPRB2 knock-out (MR K/O) mice. Furthermore, we developed cell culture assays, including MCs, keratinocytes, and macrophage cell lines, to explore the underlying mechanism and effect of GE1111 treatment. By combining in vivo and in vitro models, the present study aimed to provide a comprehensive understanding of the therapeutic potential of MRGPRX2/B2 antagonist GE1111 in LL-37-induced rosacea-like inflammation.

## Materials and methods

### Mice

Wild-type C57BL/6 N adult male mice and MR K/O adult male mice, 6 to 8 weeks old, were purchased from the Centre for Comparative Medicine Research (CCMR) of the University of Hong Kong. The animal facility is accredited by the Association for Assessment and Accreditation of Laboratory Animal Care International. The mice were housed in a controlled environment with a 24-hour light/dark cycle and provided with access to food and water. The experimental protocols involving the mice were reviewed and approved by the Committee on the Use of Live Animals in Teaching and Research (CULATR) at the University of Hong Kong (approval number 22–065). All procedures performed on the animals were conducted under anaesthesia using ketamine/xylazine by the approved CULATR protocol.

### LL-37-induced mouse model of rosacea

**Fig. 1:**
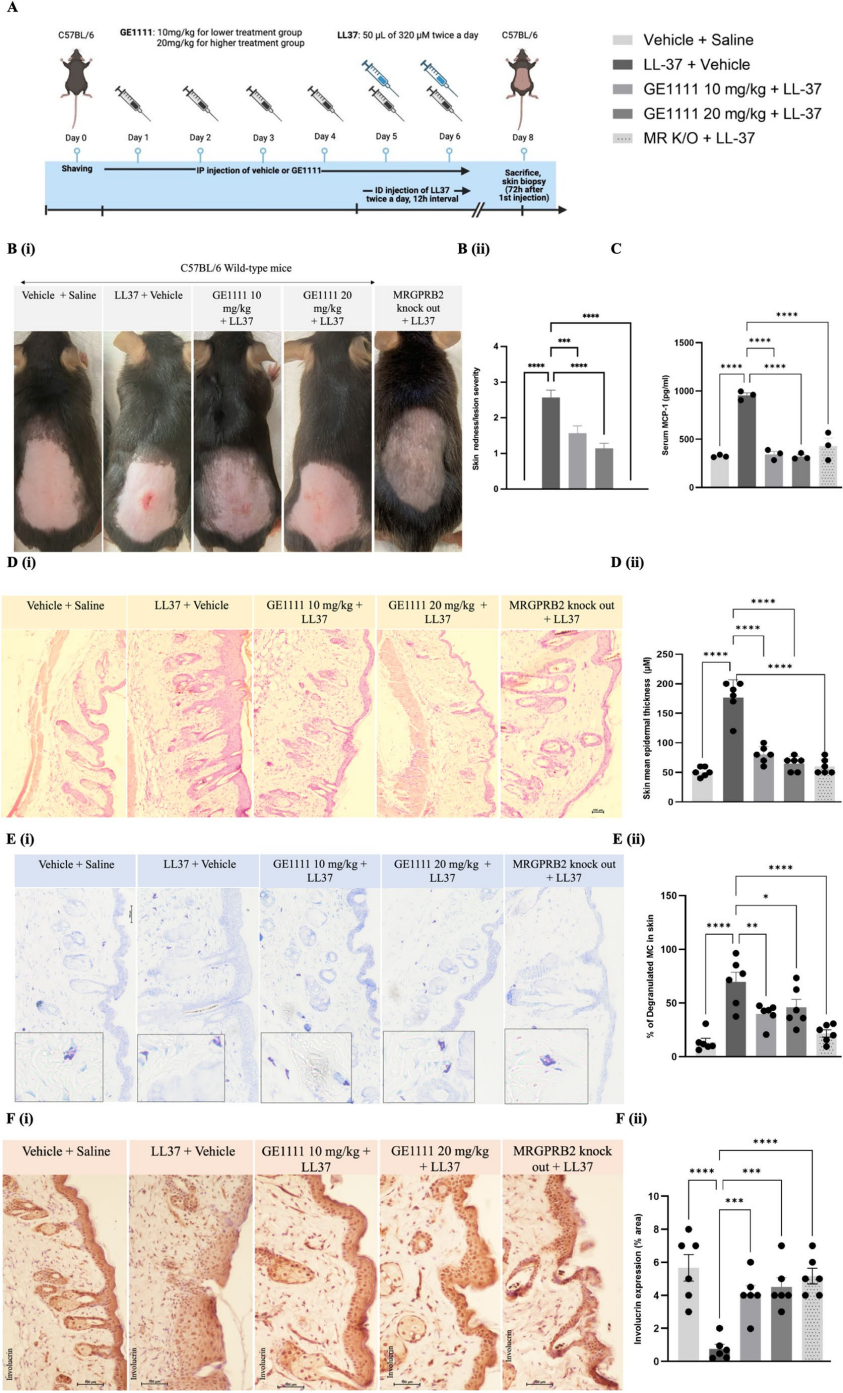
MRGPRX2/B2 antagonist GE1111 ameliorated LL-37-induced rosacea-like inflammation symptoms in mice. **A** The schematic diagram shows the LL-37-induced rosacea-like inflammation experimental design. Male C57BL/6 mice aged 6–8 weeks and MR K/O (*n* = 6) were shaved and divided into different experimental groups randomly. Mice were treated with a vehicle or GE1111 for four consecutive days. On days 5 and 6, mice were injected with 50 µl of 320µM LL-37 twice a day while vehicle and GE1111 treatment was also continued for days 5 and 6 (**B** (i)) Representative images of the mice’s skin on Day 8. (B (ii)) Grading of mouse skin redness/lesion on a scale of 0–3 (absent, mild, moderate, or severe) **C** Quantification of serum MCP-1 in the experimental mice by ELISA kit. **D** Representative images of (i) hematoxylin and eosin (H&E) staining and (ii) epidermal thickness of mice skin and **E** (i) Toluidine Blue staining of mouse skin tissue and quantitative measure of (ii) % degranulated MCs **F** (i) Representative IHC images of involucrin expression in the skin epidermis and (ii) its quantitative expression by Image J. Data from 6 mice are shown as mean ± SEM. Statistical significance was determined by one-way ANOVA and Tukey’s multiple comparisons post-hoc test: *P < 0.05, **P < 0.01, ***P < 0.001, ****P < 0.0001

**Fig. 2:**
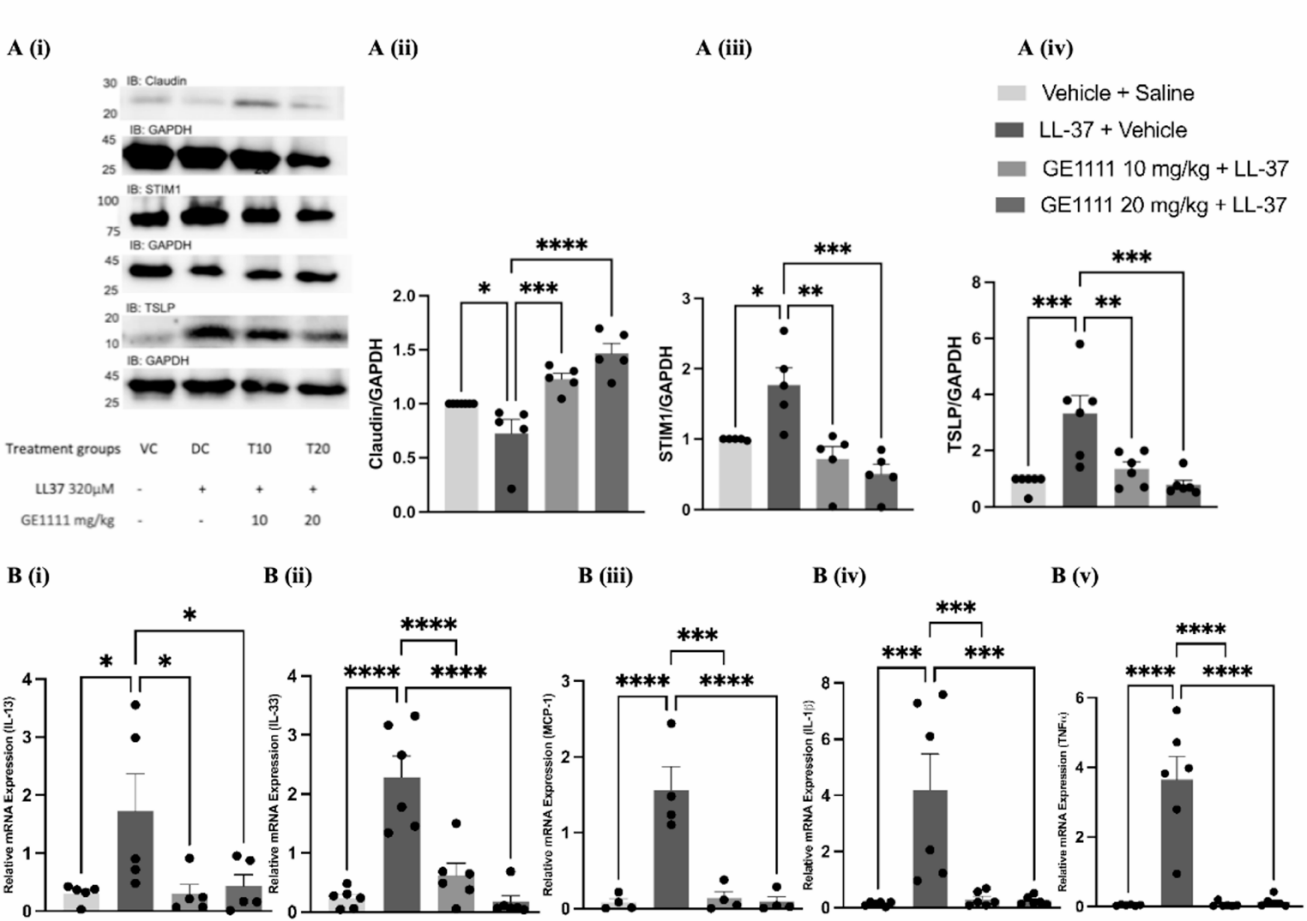
GE1111 treated mice showed significant skin integrity protection and reduced inflammatory cytokine gene and protein expression in LL-37-induced rosacea-like inflammation mice. **A** (i) Representative western blot images of claudin-1, STIM1, and TSLP protein expression in mice skin. Bar graphs representing the quantitative expression of (ii) claudin-1, (iii) STIM1, and (iv) TSLP. **B** Bar graphs representing quantification of gene expression of (i) IL-13, (ii) IL-33, (iii) MCP-1, (iv) IL-1ß, (v) TNF-α. LL37 induced rosacea like inflammation mice showed a marked increase in the all these inflammatory mediators while GE1111 showed a dose-dependent decrease in the expression of these mediators. Data from 3–7 independent experiments are shown as mean ± SEM. Statistical significance was determined by one-way ANOVA; *P < 0.05, **P < 0.01, ***P < 0.001, ****P < 0.0001

For the in vivo animal model, we used wild-type and MR K/O mice, divided randomly according to their body weight into 5 experimental groups (6 mice each). A schematic diagram outlining the experimental protocol is presented in Fig. 1A. The experimental groups were vehicle + saline (vehicle control), LL-37 treatment (disease control), lower GE1111 treatment group (T10), higher GE1111 treatment group (T20), and LL-37-MR K/O group. A total of 8 days of experimental protocol was followed briefly; on day 0, the dorsal skin area of experimental mice was shaved before LL-37/saline injection. From day 1 to day 6, 0.01% DMSO in PBS was injected via intraperitoneal injection to vehicle control, LL-37-induced disease control, and MR K/O groups. In contrast, GE1111 was injected intraperitoneally into T10 and T20 groups at a dose of 10 mg/kg and 20 mg/kg, respectively. On days 5 and 6, 50 µL of 320 µM LL-37 was injected intradermally twice a day with 12-hour intervals to LL-37 disease control, T10, T20, and MR K/O mice, while vehicle control mice were intradermally injected with vehicle. On day 8 (after 72 h of the first intradermal injection of LL-37), the severity of redness/skin lesions was graded as absent, mild, moderate, or severe (0–3). Mice were sacrificed, and the skin and blood were collected for downstream analysis (Fig. 2).

### MRGPRX2/B2 agonist C48/80 induced mouse model of itch

**Fig. 3:**
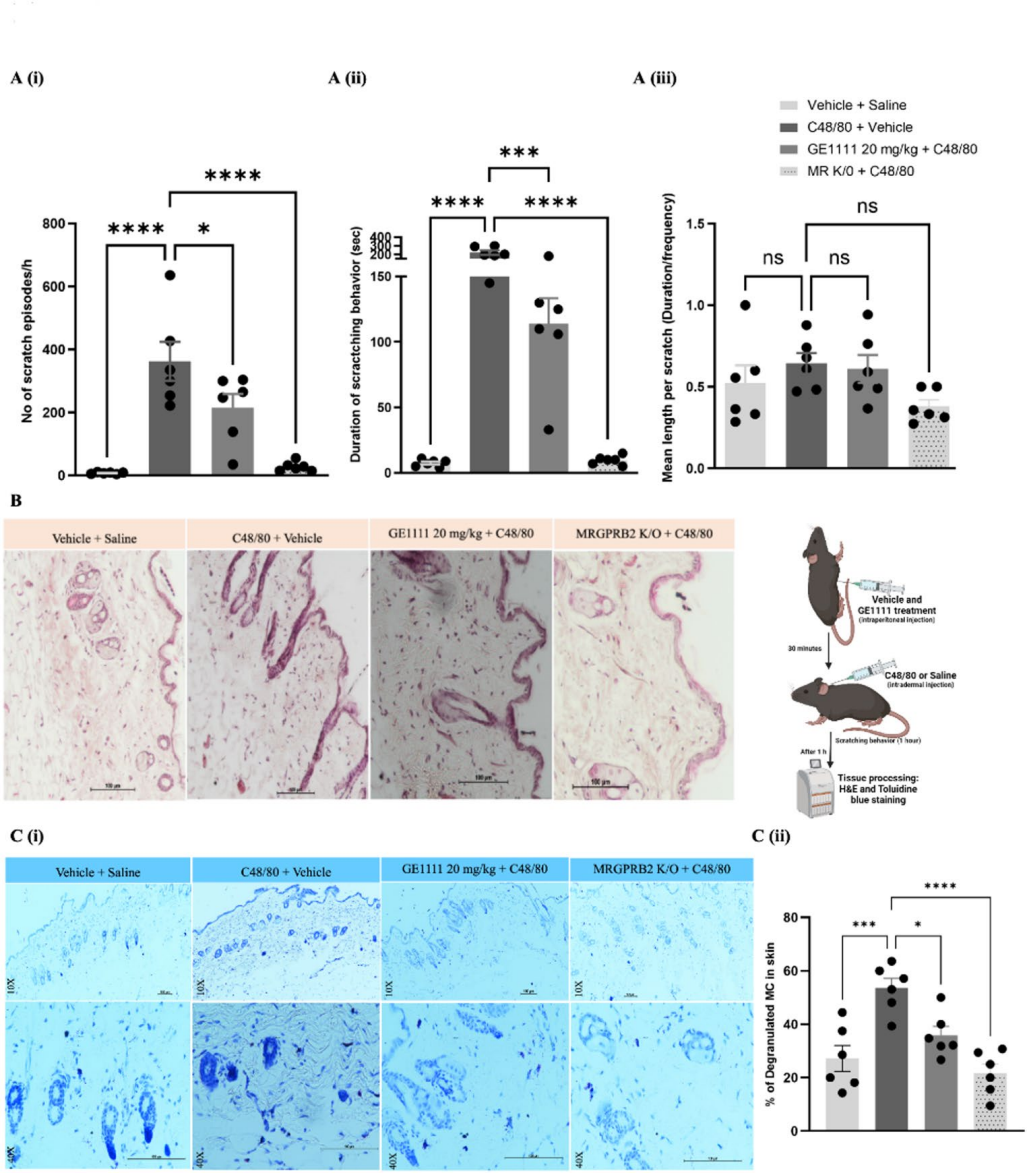
GE1111 treatment reduced the frequency and duration of scratch behaviour and number of degranulated MCs in Compound 48/80 (C48/80) induced itch mice model. **A** (i) Bar graph showing the number of scratch episodes in mice in an hour. (ii) Total time spent on scratching by mice (iii) Mean length of scratch in mice. **B** Representative image of H&E staining of skin tissue of mice and experimental design of C48/80 induced itch model. **C** Representative 20X and 40X images of Toluidine blue staining of skin tissue of mice. (ii) % Degranulated MCs in the skin tissue of mice calculated by dividing degranulated MCs by the total number of MCs. Data from 6–8 mice in each group are shown as mean ± SEM. Statistical significance was determined by one-way ANOVA; *P < 0.05, **P < 0.01, ***P < 0.001, ****P < 0.0001

We used wild-type C57BL/6 N and MR K/O mice for itch behavioral assay. Mice were acclimatised for 2–4 days before the experiment, and GE1111 (20 mg/kg body weight) was administered intraperitoneally 30 min before C48/80 injection. C48/80 (50 µL of 10 µg) was injected subcutaneously into the nape of the neck of mice, and scratching behaviour was observed for 1 h. A bout of scratching was defined as a hindpaw-directed continuous scratching movement at the C48/80 injection site. Scratching behaviour was quantified by counting the number of scratching bouts during the 1 h observation period and scored manually [20]. A schematic diagram of the experimental protocol is given on the left panel of Fig. 3B. C48/80 was selected as it activates MRGPRX2, mimicking non-histaminergic itch relevant to rosacea via mast cell degranulation [17, 20].

### Enzyme-linked immunosorbent assay (ELISA)

The MCP-1 levels in the blood of the experimental mice were evaluated using the MCP-1 ELISA kit manufactured by Thermo Fisher Scientific (Catalog number BMS6005). The 4 times dilution was done for serum samples, and the assay followed the instructions.

### Histology (H&E and toluidine blue staining)

Mouse skin and ear samples were fixed in 10% buffered formalin and were dehydrated using a series of ethanol concentrations ranging from 30 to 95%. Subsequently, these samples were embedded in paraffin blocks sectioned at a thickness of 5 μm and stained with H&E and toluidine blue staining according to our previous method [18]. Images of the sections were captured using a Nikon Eclipse Ni-U upright microscope equipped with a Nikon DS-Ri2 microscopic camera (Nikon, Tokyo, Japan) at both 10X and 20X magnifications.

### Immunohistochemistry

Paraffin sections of the skin tissues were used to analyse the expression of the tight junction protein Involucrin. DAB Substrate Kit, Peroxidase (HRP), with Nickel (3.3′-diaminobenzidine), was used (SK-4100) for immunohistochemistry as per the manufacturer’s instructions. ImageJ software was used to quantitatively analyse the stained sections [21].

### In vitro cell culture models

Chronic skin inflammatory conditions involve a complex interplay of skin and immune cells, such as MCs, keratinocytes, and macrophages. To mimic this environment in vitro, we designed and developed a model involving LAD-2 MCs, HaCaT keratinocytes, and RAW 264.7 macrophage cell line. LAD-2 cells were cultured in StemPro-34 medium supplemented with 10 mL/L Stem Pro nutritional supplements, penicillin-streptomycin (1:100), 2 mmol/L glutamine and 100ng/mL human stem cell factor and incubated at 37 °C in 5% CO_2_ incubator. The cell numbers were observed regularly, considering their doubling time. HEK-293, HaCaT and RAW 264.7 macrophage cell lines were kept in Dulbecco’s modified Eagle Medium (DMEM) supplemented with 10% Fetal Bovine Serum (FBS) and penicillin-streptomycin (1:100). We looked at the crucial proteins and genes involved in rosacea via different techniques. To investigate the impact of MRGPRX2 on the interaction between MCs and other adjacent cells (keratinocytes and macrophages), we used a potent MRGPRX2 agonist LL-37 and novel small molecule MRGPRX2 antagonist GE1111. Briefly, HaCaT keratinocytes were seeded at a density of 3 × 10^5^ cells/well (2 mL) in a six-well plate and 5 × 10^4^ cells/mL (400 µL) in ibidi 8-well Chamber, removable slides (Catalog number: 80841). Cells were incubated overnight at 37 °C in 5% CO_2_ incubator. On the second day, LAD-2 cells were seeded at a density of 1.0 × 10^6^ cells/well (800 µL) and treated with either 0.1% DMSO or 50 µM GE1111 (100 µL) for 30 min, followed by treatment with either naive media or 2.2 µM (10 µg/mL) of LL-37 (100 µL) for 2 h. After 2 h, the MCs were collected and centrifugated at 500 g for 5 minutes to isolate the MCs supernatant while MCs lysate was harvested for gene or protein expression analysis. The isolated supernatant (800 µL) was then transferred to keratinocytes and incubated for an additional 2 h. After 2 h incubation period, keratinocytes were harvested for subsequent gene or protein analysis. For immunofluorescence assay, 150 µL of LAD-2 MCs supernatant was transferred onto HaCaT keratinocytes and incubated for 14–16 h at 37 °C in 5% CO_2_ incubator.

### Mast cell degranulation assay

We performed a β-hexosaminidase release assay according to our previously modified method [18]. For IC_50_ determination, MCs were treated with graded concentrations of GE1111 and stimulated with a fixed 90% effective concentration (IC90; ~5 µM) of LL-37. For EC_50_ determination, MCs were treated with a fixed concentration of GE1111 (5 µM) or buffer and stimulated with graded concentrations of LL-37 (0-100 µM). PNAG (4-Nitrophenyl N-acetyl-β-D-glucosaminide, 1.3 mg/ml) was added to each well of supernatant and lysate, and cells were incubated for 90 min at 37 °C (without CO2). The reaction was stopped by adding 50 µL of 0.4 M Glycine buffer. The extent of yellow color was measured at 405 nm using Victor 4X plate reader (PerkinElmer), and % MCs degranulation (% β-hexosaminidase release) was calculated.

### Calcium flux assay

To evaluate and validate the effect of GE1111 on LL-37 mediated MRGPRX2 activation, we previously performed β-arrestin PRESTO TANGO assay. However, we were not able to find any change in the luminescence in the β-arrestin PRESTO TANGO assay for LL-37. Therefore, we designed a gene knock-in experiment by using a calcium flux assay in the MRGPRX2 transfected HEK-293 cell line. On day 1 of the experiment, HEK-293 cells were seeded at 1 × 10^6^ cells per 100 mm cell culture dish overnight. On day 2, HEK-293 cells were transfected with MRGPRX2 DNA or mock-transfected using the Lipofectamine transfection reagent (Thermo Fischer Scientific) and incubated for 24 h. On day 3, MRGPRX2 transfected cells were transferred into 96-well black wall cell culture plates at a density of 2 × 10^4^cells/well/80 µL (Thermo Fischer Scientific). On day 4, 40 µL of the 2X Fluo-4 NW calcium loading solution and or buffer was added (Molecular Probe, Invitrogen, USA). Cells were incubated for 30 min at 37 °C in a 5% CO2 incubator, followed by treatment with GE1111 (in the same manner as MC degranulation assay) and 30-minute incubation. After one hour of incubation, a graded concentration of LL-37 or buffer was added and the fluorescence intensity was measured at an excitation wavelength of 494 nm and an emission wavelength of 516 nm using Victor 4X plate reader (PerkinElmer) for 150 s. The ΔF was calculated by subtracting the maximum wavelength (after adding LL-37) from minimum fluorescence (before adding LL-37) and plotted by using Graph Pad Prism software 9.3.1.

### Immunofluorescence assay

**Fig. 4:**
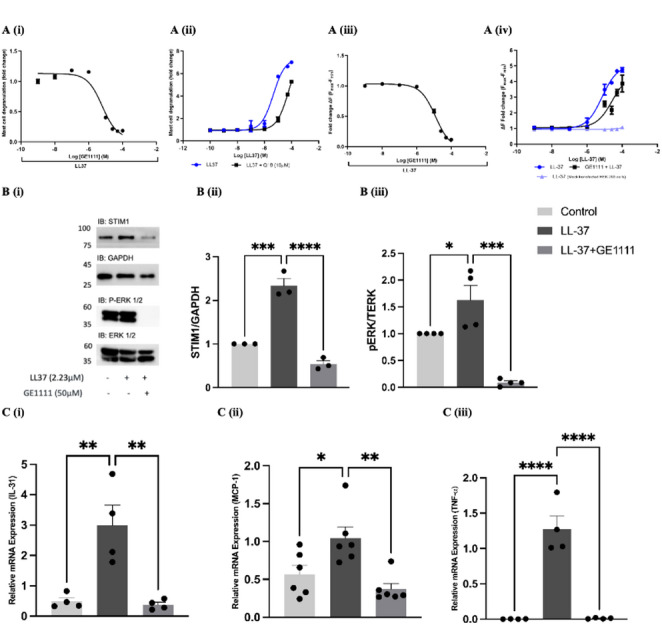
Effect of the MRGPRX2/B2 antagonist GE1111 on LL-37 induced MCs degranulation, MRGPRX2 mediated Calcium flux, downstream signalling pathways and inflammatory cytokines in LAD-2 MCs. (**A** (i & ii)) IC_50_ and EC_50_ concentration-response curves of GE1111 and LL-37 were performed by MCs degranulation assay. (A (iii & iv)) Calcium flux assay on MRGPRX2-transfected HEK-293 cells to plot the concentration-response curve of GE1111 and LL-37. **B** (i) Representative Western blots of ERK 1/2 (P-ERK 1/2) and STIM1 protein expression after 2 h of incubation with LL-37 in the presence and absence of GE1111. Bar graphs representing the relative band intensities for *(ii)* ERK 1/2 and *(iii)* STIM1 **C** Bar graphs representing relative mRNA expression of *(i)* IL-31, *(ii)* MCP-1, and *(iii)* TNF-α. Data from 3–5 independent experiments are shown as mean ± SEM (n = at least 4 unless otherwise stated). Statistical significance was determined by one-way ANOVA and Tukey’s multiple comparisons post-hoc test; *P < 0.05, **P < 0.01, ***P < 0.001, ****P < 0.0001

The experimental design was described in the above paragraph and in Fig. 4A. Briefly, HaCaT cells were incubated in the presence and absence of different MCs supernatant groups for a duration of 12–14 h. Fixed keratinocytes were blocked using 5% BSA for one hour at room temperature, followed by incubation with primary antibodies specifically targeting claudin-1 (Ab15098) and TSLP (PA5-89013) for 2 h at room temperature. Alexa Fluor 594 donkey anti-Rabbit IgG (red fluorescence) was used to detect claudin-1, and Alexa Fluor 488 donkey anti-Rabbit IgG (green fluorescence) was used to detect TSLP. The immunofluorescence staining was observed and captured using a Fluorescent Microscope equipped with a Nikon DS-Ri2 camera. Images were acquired at 20X magnificent levels to examine the cellular localization and expression levels of TSLP and claudin-1 within the HaCaT keratinocytes.

### Western blotting

The in-vitro samples were subjected to lysis using RIPA lysis, extraction buffer, and cell scrapers, along with a phosphatase and protease inhibitor cocktail (Thermo-Fisher). The protein concentration of the samples was determined using the Bradford protein assay (Thermo-Fisher). To prepare the SDS-PAGE gels, 10%, and 15% acrylamide resolving gels (pH 8.8) were manually cast, with a 4% acrylamide stacking gel. Each well of the gel was loaded with 30 µg of protein, and the gels were run at 100 V for 2 h in a running buffer for gel electrophoresis. Primary antibodies specific to the proteins of interest, including STIM1 (CST-D88E10), TSLP (PA5-89013), claudin-1 (Ab15098), GAPDH (CST-141CO), phospho-ERK 1/2 (CST-4370T) and ERK 1/2 (CST-4695T) were prepared at a dilution of 1:1000 in 5% BSA and incubated with the membrane overnight at 4 °C. Following the primary antibody incubation, membranes were washed 3 times, 15 min each with TBS-T. Secondary antibodies such as Goat anti-rabbit IgG (CST-7074), diluted at 1:5000–10,000 in 5% BSA, were added to the membrane and incubated for 2 h at room temperature. After another 3 rounds of washing, immunoreactive proteins were detected using UVITEC Alliance LD with SuperSignal Technology. The band intensities were quantified and analysed using ImageJ software [21].

### RNA isolation and quantitative RT‒PCR (RT-qPCR).

Alongside protein analysis, RT-qPCR was utilized to analyze the expression levels of various inflammatory cytokines, chemokines, and mediators in LAD-2 human MCs, HaCaT keratinocytes, and mouse skin samples. The RNA extraction process was carried out following the protocol of the FastPure Cell/Tissue Total RNA Isolation Kit V2 RC112 (Vazyme). In the case of mouse samples, additional homogenization was performed through agitation using the Fisherbrand™ Bead Homogenizer Accessory and Buffer RL from the FastPure Kit. Skin samples were first cut with sterilized scissors, then homogenized using an agitator for 20 min. To convert RNA into cDNA, the HiScript II 1st Strand cDNA Synthesis Kit R211 (Vazyme) was employed, following the instructions provided with the kit. Subsequently, PCR was conducted using the ChamQ SYBR Color RT-qPCR Master Mix (High ROX Premixed) Q441 (Vazyme) according to the kit’s protocol. The primers used for the PCR amplification, along with their corresponding sequences, can be found in Table 1.

**Table 1:** Primer sequence for human and mouse inflammatory cytokine genes

Human gene	Forward (5′-3′)	Reverse (5′3′)
IL-13	ACGGTCATTGCTCTCACTTGCC	CTGTCAGGTTGATGCTCCATACC
IL-31	GCCCAGCCGCCAAAC	GCTGTCTGATTGTCTTGAGATATGC
IL-17	AATCTCCACCGCAATGAGGA	ACGTTCCCATCAGCGTTG
IL-8	AAGAGAGCTCTGTCTGGACC	GATATTCTCTTGGCCCTTGG
MCP-1	CAGCCAGATGCAATCAATGCC	TGGAATCCTGAACCCACTTCT
TNF-α	GGTGCCTATGTCTCAGCCTCTT	GGTGCCTATGTCTCAGCCTCTT

Mouse gene	Forward (5′-3′)	Reverse (5′3′)
MCP-1	GTTGGCTCAGCCAGATGCA	AGCCTACTCATTGGGATCATCTTG
IL-13	AACGGCAGCATGGTATGGAGTG	TGGGTCCTGTAGATGGCATTGC
IL-33	CTACTGCATGAGACTCCGTTCTG	AGAATCCCGTGGATAGGCAGAG
IL-1ß	TGGACCTTCCAGGATGAGGACA	GTTCATCTCGGAGCCTGTAGTG
TNF-α	GGTGCCTATGTCTCAGCCTCTT	GCCATAGAACTGATGAGAGGGAG

### Statistical analysis

Statistical analysis was performed using GraphPad Prism 9 software (GraphPad Software, La Jolla, California) or Microsoft Excel (Microsoft, Redmond, Washington) software. All the graphs were plotted by GraphPad Prism 9.3.1, and the data are presented as means ± SEMs of at least 3 independent experiments unless otherwise specified. The P values are represented by * and shown on top of the corresponding columns, as determined by 1-way ANOVA followed by Tukey’s multiple comparisons post-hoc test.

## Results

### MRGPRX2/B2 antagonist GE1111 treatment reduced the rosacea symptoms and inflammatory mediators in LL-37-induced rosacea-like inflammation in mice

We evaluated the effect of MRGPRX2/B2 antagonist GE1111 on a mice model of LL-37-induced rosacea-like inflammation and also validated the effect by using MR K/O mice. The experimental design and representative images of lesions are shown in Fig. 1A and B, respectively. Figure 1B (ii) showed the skin redness/lesion severity in different experimental groups. LL-37-treated wild-type mice exhibited significant redness/small lesions and higher scores, while the symptoms were reduced in GE1111-treated mice and absent in vehicle control and MR K/O mice. There were no phenotypic changes in MR K/O vehicle control mice (vehicle + saline, data not shown). Treatment with GE1111 reduced redness and lesions (Fig. 1B i & ii). The serum MCP-1 was significantly elevated in LL-37-treated mice compared to vehicle control and LL-37 treated MR K/O mice (Fig. 1C). MCP-1 level was reduced in mice treated with GE1111. Furthermore, the epidermal thickness was significantly increased in LL-37-treated mice compared to the vehicle control mice and LL-37-treated MR K/O mice (Fig. 1D). Mice treated with GE1111 showed a significant reduction in epidermal thickness. We also looked at the % degranulated MCs in the skin tissue and found a significant increase in degranulated MCs around the epidermal and dermal region in LL-37-treated mice. Mice treated with 10 mg/kg and 20 mg/kg GE1111 showed a significant reduction in the degranulated MCs as compared to LL-37-treated mice (Fig. 1E (i & ii)). The skin integrity in LL-37-treated mice was compromised as evidenced by significantly lower expression of the epidermal barrier protein Involucrin compared to control and LL-37 treated MR K/O mice (Fig. 1F). Mice treated with GE1111 demonstrated significant protection of this tight junction protein (Fig. 1F (ii)). No overt signs of irritation or toxicity were noted in GE1111-treated mice during the study period.

### GE1111-treatment reduced inflammatory cytokines and downstream signalling pathways in LL-37-induced rosacea-like inflammation in mice

We measured the inflammatory surge and MRGPRX2/B2 signalling pathway in vivo model of rosacea. We observed a significant reduction in claudin-1 and increased TSLP protein expression in LL-37-treated mice compared to vehicle control mice, indicating a disrupted skin barrier and inflammation (Fig. 2A). These effects were reversed with GE1111 treatment, showing decreases in TSLP and restoration of claudin-1 levels. Additionally, STIM1 protein expression, crucial for MC activation via MRGPRX2/B2 signalling, was elevated in LL-37-treated mice, which was reduced with GE1111 treatment (Fig. 2A (iii)).

Furthermore, gene expression analysis revealed elevated levels of inflammatory cytokines IL-13, IL-33, MCP-1, IL-1β, and TNF-α in LL-37-treated mice, consistent with rosacea’s symptoms and serum MCP-1 level. GE1111 treatment significantly lowered the gene expression of these cytokines (Fig. 2B).

### GE1111 treatment ameliorates compound 48/80 induced itch in mice

Itch is one of the common symptoms of rosacea and other chronic skin inflammatory conditions. To assess the effect of GE1111 on itch, we used a mouse model using synthetic MRGPRX2/B2 agonist C48/80. Previous studies have shown the non-histaminergic itch pathway involvement via MRGPRX2/B2 mediated MCs activation [20]. Previously we showed the antagonistic effect of GE1111 against C48/80 induced MRGPRX2-MCs degranulation and acute allergic reactions [18]. C48/80 significantly increased both the number and duration of scratching in wild-type mice as compared to vehicle control and C48/80 treated MR K/O mice. Treatment with GE1111 reduced the number and duration of scratching episodes but did not affect the average length of scratches. Skin histology showed no changes in epidermal thickness but showed a significant increase in degranulated MCs in C48/80 treated mice compared to vehicle control and C48/80 treated MR K/O mice which was significantly reduced by GE1111 treatment, indicating its effectiveness in lowering itch by inhibiting MCs degranulation (Fig. 3C (i & ii)).

### GE1111 antagonised LL-37-induced MCs degranulation, inflammatory gene and downstream signalling proteins in MCs

To investigate the underlying molecular mechanism of GE1111 in reducing rosacea symptoms and inflammation, we designed in vitro functional assays using MCs. GE1111 inhibited MCs degranulation and MRGPRX2 mediated calcium flux, evidenced by its IC_50_ values in MCs degranulation (6.271 µM) and calcium flux assays (12.31 µM) with LL-37 (Fig. 4A (i and iii)). In addition, GE1111 significantly increased the EC_50_ of LL-37 from 4.542 µM to 60.320 µM in the MCs degranulation assay and the EC_50_ of LL-37 from 7.163µM to 26.680µM in the calcium flux assay, indicating its inhibitory effect on MCs activation by LL-37 (Fig. 4A (ii and iv)). Furthermore, we found that GE1111 inhibited the activation pathway of MRGPRX2-MCs by reducing the expression of STIM1 and phosphorylation of ERK _1/2_ (Fig. 4B). Moreover, GE1111 treatment significantly decreased the gene expression of inflammatory cytokines, including IL-31, MCP-1, and TNF-α in MCs, which are critical in rosacea’s inflammatory response (Fig. 4C).

### GE1111 treated MCs attenuated the expression of LL-37-induced inflammatory cytokines and restored tight junction protein in human HaCaT keratinocytes

**Fig. 5:**
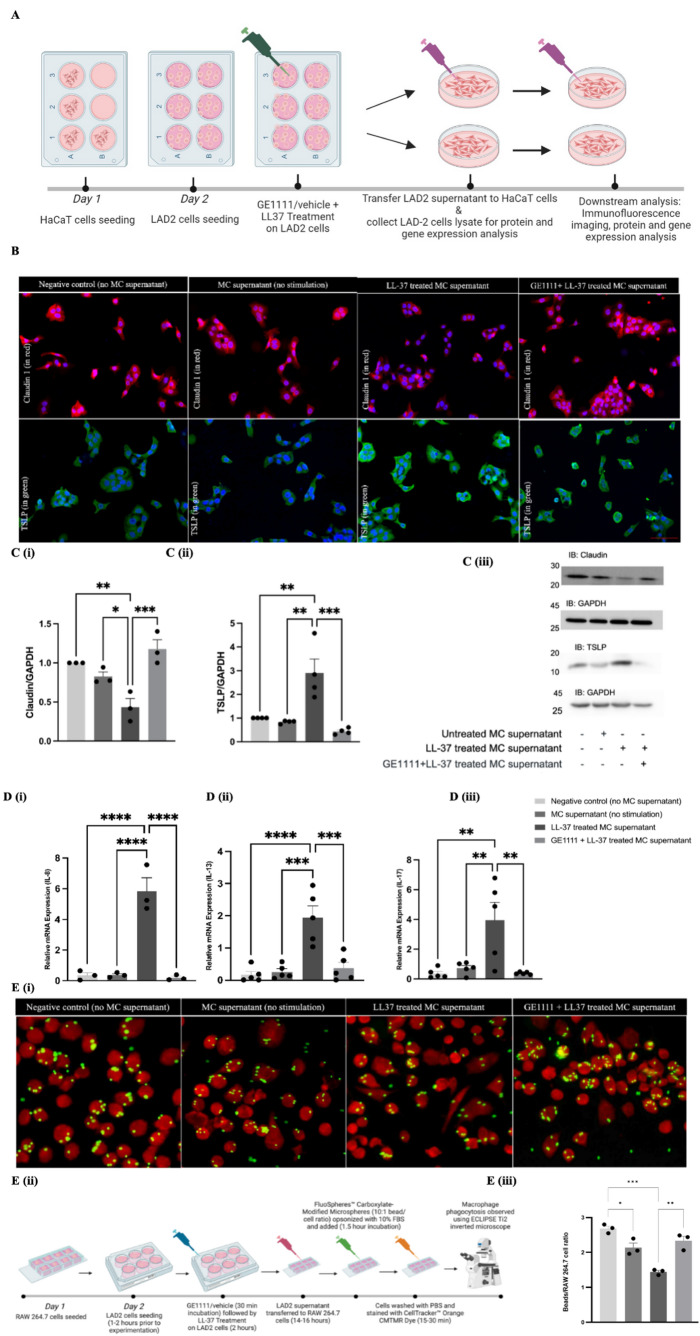
GE1111 restored the epidermal skin barrier and reduced inflammatory gene expression in HaCaT human keratinocyte cell line. Moreover, GE1111 treatment restored the phagocytotic ability of macrophages. **A** In-vitro experimental design of MCs and HaCaT keratinocyte conditioned media experiment. HaCaT human keratinocytes were treated with vehicle, naïve LAD-2 supernatant, 2.23 µM LL-37-treated LAD-2 supernatant, or 2.23µM LL-37 + 50µM GE1111-treated LAD-2 supernatant for 2 h. **B** Immunofluorescent staining of keratinocytes following incubation with mast cell supernatant or vehicle for *(i)* claudin-1 & DAPI, and *(ii)* TSLP & DAPI. **C** Bar graphs representing the relative band intensities for *(i)* claudin-1 and *(ii)* TSLP, *(iii)* Representative Western blots of claudin-1 and TSLP protein expression. **D** Bar graphs representing increase in the relative mRNA expression of *(i)* IL-8, *(ii)* IL-13, and *(iii)* IL-17 HaCaT cells incubated with LAD-2 conditioned media from different experimental groups. GE1111 treated mast cells reversed these changes **E** *(i)* Fluorescence imaging of macrophage phagocytosis. The LAD-2 MCs supernatant was isolated and transferred onto the RAW 264.7 macrophages and incubated for 12–14 h. The macrophages were then incubated with the opsonised target beads and assessed for phagocytic activity *(ii)* Schematic diagram of experimental design for MCs-macrophage-phagocytosis response *(iii)* Bar graph representing the ratio of phagocytosed beads to macrophages numbers. Data from 3–6 independent experiments are shown as mean ± SEM. Statistical significance was determined by one-way ANOVA and Tukey’s multiple comparisons post-hoc test; *P < 0.05, **P < 0.01, ***P < 0.001, ****P < 0.0001

In rosacea, immune and non-immune cells, including keratinocytes, release inflammatory cytokines, exacerbating symptoms and compromising skin integrity [22]. We investigated the interaction between MCs and keratinocytes in rosacea by using an in vitro model with LAD-2 MCs and HaCaT keratinocytes [23, 24] (Fig. 5A). Immunofluorescence results showed that LL-37 treated MCs supernatant reduced claudin 1 expression and increased TSLP expression in keratinocytes compared to the negative control (Fig. 5B). Figure 5C validated the immunofluorescence data and showed a significant decrease in claudin 1 protein expression and increased expression of TSLP in keratinocytes challenged with LL-37 treated MCs supernatant (Fig. 5C). GE1111 treatment significantly reversed the change in claudin 1 and TSLP expression. Figure 5D showed a significant increase in the gene expression of inflammatory cytokines, including IL-8, IL-13, and IL-17 in keratinocytes challenged with LL-37 treated MCs supernatant as compared to the negative control group (no MC supernatant). Furthermore, our results showed that keratinocytes treated with LL-37 + GE1111 MCs supernatant exhibited a significant reduction in the gene expression of inflammatory cytokines induced by LL-37, compared to the group treated only with LL-37 MCs supernatant IL-8, IL-13 and IL-17.

### GE1111-treated MCs restored the LL-37-induced macrophage phagocytosis

ó

In addition to MCs, macrophages are also recognised as key immune cells that play a significant role in the development of rosacea [25–27]. We hypothesized that, similar to MCs and keratinocytes, crosstalk between MCs and macrophages mediated by LL-37-MRGPRX2-MC can modulate macrophage activity. Experiments using RAW 264.7 macrophage cells (Fig. 5E) showed a significant reduction in phagocytic activity when exposed to LL-37 treated MCs supernatant, compared to a control group (Fig. 5E iii). However, this decrease in phagocytosis was reversed when macrophages were treated with a combination of LL-37 and GE1111, suggesting that GE1111 mitigates the negative effects of LL-37 on macrophage function. Additionally, a decrease in phagocytosis was observed even in macrophages treated with unstimulated MCs supernatant, indicating the possibility of spontaneous MCs degranulation without any stimulus.

## Discussion

In the present study, we investigated the efficacy of novel small molecule MRGPRX2 antagonist GE1111 by combining in vivo and in vitro experimental approaches. We utilized human cathelicidin LL-37, known to play a role in rosacea’s pathogenesis, as an MRGPRX2/B2 agonist [11, 28]. Collective evidence has shown increased MCs numbers in rosacea patients, where MCs degranulation leads to the release of multiple inflammatory mediators [29–32].

Besides observing and grading redness/lesions of the skin around the LL-37 injection area, we quantified serum MCP-1 levels in experimental mice. Grading redness/lesions of the skin is also one of the diagnostic methods clinically. This was supplemented with MCP-1 serum quantification, which is a crucial cytokine released from MCs and recruits inflammatory cells [33]. MCP-1 (monocyte chemoattractant protein-1) is a chemokine that plays a critical role in the recruitment of monocytes and macrophages to sites of inflammation. In rosacea, increased levels of MCP-1 have been associated with heightened inflammatory responses and may contribute to the pathogenesis of the disease by facilitating immune cell infiltration into the skin [22, 34–37]. Therefore, measuring MCP-1 levels provides insight into the inflammatory milieu present in rosacea. Serum MCP-1 levels correlated with skin inflammation severity, consistent with reports linking systemic MCP-1 to local rosacea pathology [22].

Mice treated with LL-37 demonstrated higher redness/lesions and significantly high MCP-1 levels compared to the vehicle control group. In contrast, GE1111 treatment resulted in a significant reduction in redness/lesions and serum MCP-1 levels. These findings provide further evidence of the potential of GE1111 in modulating the inflammatory responses associated with rosacea. Skin thickening is also a common symptom observed in rosacea patients, often caused by skin fibrosis, along with heightened expression of epidermal proteases and production of pro-inflammatory cathelicidin peptides [8, 38]. The results showed increased thickness and MC degranulation in rosacea mice induced by LL-37, significantly reduced in mice treated with GE1111, highlighting its protective role in skin inflammation.

The epidermal barrier protein Involucrin has been reported to decrease in rosacea along with the diminished skin barrier [39]. Its decreased expression in LL-37-induced rosacea-like inflammation mice was restored in GE1111-treated mice, suggesting that GE1111 can help maintain skin barrier integrity. Protein expression analysis in the rosacea lesioned skin showed a significant increase in TSLP and STIM1 expression, which are involved in the MRGPRX2/B2 signalling pathway and MCs degranulation [40, 41]. STIM1 is a calcium sensor involved in store-operated calcium entry (SOCE), which is essential for MCs degranulation via the MRGPRX2 pathway [41]. However, the heightened STIM1 expression observed in the skin tissue may not solely reflect expression in MCs. Also, the increased STIM1 expression may be contributed by keratinocytes and other structural cells within the rosacea mice rather than being exclusively associated with MCs [42]. Analysis focused on claudin-1 due to its central role in LL-37-mediated barrier disruption; filaggrin and loricrin were not assessed but are often similarly affected in rosacea models [39]. Broader barrier protein profiling (e.g., filaggrin, loricrin) was not conducted; claudin-1 was selected based on established relevance [43].

GE1111 treatment effectively ameliorated the overexpression of these signalling molecules, further supporting its ability to modulate the MRGPRX2/B2 pathway. Gene expression quantification revealed an upregulation of inflammatory cytokines IL-33, IL-13, MCP-1, and IL-1ß that have been identified in the pathogenesis of rosacea. GE1111 treatment significantly reduced the gene expression of these cytokines and indicated its potential to suppress inflammatory responses in rosacea [44–47].

Itching is one of the key symptoms of rosacea and is common in several subtypes of rosacea. We used a non-histaminergic itch model induced by compound 48/80 in mice to study the effects of GE1111 [17, 48, 49]. We observed that GE1111 treatment significantly reduced C48/80 induced scratching behavior and MCs degranulation, aligning with previous findings on its inhibitory effects on C48/80 -MRGPRX2-MCs degranulation [18]. Our preliminary findings align with previous studies using C48/80-induced itch and MRGPRX2/B2 involvement [17, 49]. Additionally, we observed a correlation between MCs degranulation and itching behavior which was reduced by GE1111 treatment. On comparing the intensity of degranulated MCs in the itch and rosacea model we observed that LL-37 induced more prominent MCs degranulation as compared to compound 48/80. Possible reasons might be the difference in the binding intensity and efficacy of these agonists for MRGPRX2 and single (compound 48/80) vs. multiple injection (LL-37) of agonists. The C48/80 model’s relevance to rosacea pruritus lies in its MRGPRX2-dependent pathway, supported by studies linking compound 48/80 to mast cell-mediated itch in inflammatory skin conditions [50]. Results indicate GE1111’s effects on MRGPRX2 inhibition, which contributes to anti-pruritic outcomes; alternative itch models (e.g., acute/chronic) could further validate broad anti-itching efficacy [51]. As the model demonstrates MRGPRX2-specific effects; broader pruritus models are needed for comprehensive anti-itch assessment.

Another significant observation from the two disease models in our study was the differing effects of GE1111 and the MRGPRB2 gene knockout. We found that GE1111 had a more pronounced impact on rosacea compared to the itch model. Conversely, the MRGPRB2 knockout mice exhibited a stronger effect in the itch model than in the rosacea model. GE1111 demonstrated a more pronounced effect on rosacea, likely due to its ability to inhibit inflammatory cytokines and restore skin integrity, which are critical for managing this chronic inflammatory condition. In contrast, MRGPRB2 knockout mice exhibited stronger responses in the itch model, suggesting that mast cell activation pathways may differ between these conditions [5, 20]. These findings underscore the complex interplay of immune responses and mast cell activation in the pathology of rosacea and itch, emphasizing the need for more studies to understand the complex interaction.

We used MCs, keratinocytes and macrophages to elucidate the underlying molecular mechanism. We explored GE1111’s impact on the LL-37-MRGPRX2 mediated MCs degranulation and inflammation [52–54]. GE1111 effectively inhibited LL-37-induced MCs degranulation, calcium flux, downstream signalling, and inflammatory cytokine gene expression. This aligns with our previous findings on the broad antagonistic activity of GE1111 [18]. Activation of MRGPRX2 in MCs triggers a cascade of events, including the activation of ERK _1/2_ and STIM1, leading to the release of granules and subsequent MC degranulation. GE1111 treated MCs demonstrated a significant decrease in phosphorylated ERK 1/2 and STIM1 protein expression. Furthermore, GE1111 reduced the LL-37-induced gene expression of MCP-1, IL-31, and TNF-α in MCs, which upregulated in rosacea [22, 34–37].

Furthermore, we used the MCs conditioned media to investigate the interaction of MCs with keratinocytes and macrophages [12, 33]. MRGPRX2-mediated mast cell activation by LL-37 may lead to the release of various proteases, histamine, and cytokines that interact with both immune and non-immune cells, thereby altering the immune microenvironment [5, 55]. However, besides quantifying gene expression of certain cytokines in LL-37-activated MCs, we did not look into the expression of other inflammatory mediators. We demonstrated that keratinocytes exhibited increased protein and gene expression of TSLP when incubated with LL-37 treated MCs, suggesting a correlation with our in vivo observations in a rosacea model. TSLP, a key type 2 cytokine implicated in chronic skin inflammation, has recently been linked to MRGPRX2 activation, which is also associated with the pathogenesis of rosacea [23, 47, 56, 57]. MRGPRX2 antagonist GE1111 treatment significantly reduced the LL-37-induced gene and protein expression of TSLP. Moreover, we observed that GE1111 effectively preserved the expression of claudin 1, a critical tight junction protein essential for maintaining epidermal barrier integrity, which is often compromised in rosacea, leading to aggravated symptoms and potential secondary infections [58]. To further investigate the efficacy of GE1111 in reducing inflammation, we measured the gene expression of shortlisted cytokines such as IL-8, IL-13, and IL-17 in HaCaT keratinocytes. These cytokines demonstrated a higher expression in rosacea and other chronic skin allergies, indicating that GE1111 might reduce inflammation by lowering the number and quantity of inflammatory mediators in MCs supernatant, thus decreasing keratinocyte activation [44–47]. In addition, we looked at the interaction between MCs and macrophages. In our previous study with CST-14, we found a decreased response of macrophages therefore we hypothesized a similar response with LL-37 in the context of rosacea [59]. We showed that macrophages challenged with LL-37 induced MCs conditioned media reduced the ability of macrophage phagocytosis, suggesting a modulation and dysregulation of the phagocytotic ability of the macrophages [25–27]. MRGPRX2 antagonist GE1111 significantly restored the LL-37-induced dysregulated phagocytosis ability of macrophages. Our findings showed the ability of MCs-MRGPRX2 to modulation of keratinocytes and macrophages which can be further explored to pinpoint the molecular mechanism.

A limitation of the current study isthe lack of pharmacokinetic data, safety profile, and clinical data supporting the potential therapeutic role of GE1111 in rosacea. And the absence of dedicated histopathological assessments for GE1111-induced skin changes or dose-response proliferation assays on mast cells, HaCaT cells, and macrophages. However, no adverse skin effects were observed in treated cohorts, aligning with our previous study, we reported the MRGPRX2 specificity of GE1111 and that there was no cytotoxicity in the cell-based MTT assay [18]. Protein-level MCP-1 validation in skin tissue (e.g., via WB or IHC) was not performed; serum ELISA was used as a proxy for systemic inflammation. Literature supports serum MCP-1 as reflective of local skin changes in rosacea models, though correlations may vary [37]. Tissue-specific protein analysis is a priority for future studies incorporating acute and chronic toxicity studies and clinical specimens are required to support the translational potential of MRGPRX2/B2 antagonist GE1111 in rosacea.

## Conclusion

Our study provided in-depth phenotypic and mechanistic evidence on the protective effect of novel MRGPRX2/B2 antagonist GE1111 in attenuating LL-37-induced rosacea-like inflammation and compound 48/80-induced itch in mice. These findings can be exploited to further the investigation of GE1111 in rosacea and other inflammatory skin conditions.

## Data Availability

No datasets were generated or analysed during the current study.
